# Statistical analysis reveals the onset of synchrony in sparse swarms of *Photinus knulli* fireflies

**DOI:** 10.1098/rsif.2022.0007

**Published:** 2022-03-23

**Authors:** Raphaël Sarfati, Laura Gaudette, Joseph M. Cicero, Orit Peleg

**Affiliations:** ^1^ BioFrontiers Institute, University of Colorado, Boulder, CO, USA; ^2^ Department of Computer Science, University of Colorado, Boulder, CO, USA; ^3^ Department of Ecology and Evolutionary Biology, University of Colorado, Boulder, CO, USA; ^4^ Department of Physics, University of Colorado, Boulder, CO, USA; ^5^ Department of Applied Math, University of Colorado, Boulder, CO, USA; ^6^ McGuire Center for Lepidoptera and Biodiversity, Florida Museum of Natural History, Gainesville, FL, USA; ^7^ No affiliation; ^8^ Santa Fe Institute, Santa Fe, NM, USA

**Keywords:** fireflies, synchronization, statistical analysis, collective behaviour

## Abstract

Flash synchrony within firefly swarms is an elegant but elusive manifestation of collective animal behaviour. It has been observed, and sometimes demonstrated, in a few populations across the world, but exactly which species are capable of large-scale synchronization remains unclear, especially for low-density swarms. The underlying question which we address here is: how does one qualify a collective flashing display as synchronous, given that the only information available is the time and location of flashes? We propose different statistical approaches and apply them to high-resolution stereoscopic video recordings of the collective flashing of *Photinus knulli* fireflies, hence establishing the occurrence of synchrony in this species. These results substantiate detailed visual observations published in the early 1980s and made at the same experimental site: Peña Blanca Canyon, Coronado National Forest, AZ, USA. We also remark that *P. knulli*’s collective flashing patterns mirror those observed in *Photinus carolinus* fireflies in the Eastern USA, consisting of synchronous flashes in periodic bursts with rapid accretion and quick decay.

## 1. Introduction

Many animal species are capable of, and benefit from, behaving collectively, from insects forming rigid aggregates, such as ants and bees, to large mammals migrating as herds over thousands of kilometres. Although in some cases the emergence of collective dynamics is intuitively evident, for example in collective turns of flocking birds or swirling schools of fish, in other instances characterizing the ensemble structure or dynamics as collective requires a much finer analysis than simple visual observations. This is the case, for example, in disorganized midge swarms [1]. In fact, despite a growing interest for large-scale patterns in animal groups, a clear definition of which behaviour qualifies as collective is still lacking [2].

To address this broad and complex question, it may be easier to start with a simple subset of collective behaviour: synchrony. Animal synchronization manifests itself in many different ways and across various time scales [3], and it is certainly a signature of how social interactions produce system-wide patterns. An inspiring and readily accessible example of biological synchrony is seen sometimes on summer nights in firefly swarms, when most flashes occur at specific instants.

Firefly flashing is primarily a courtship dialogue. In some species, advertising males flash in unison while females respond independently. Initially observed in Southeast Asia, synchronous fireflies were first reported in North America in the 1910s [4]. Observations were rare and sporadic, and often received with scepticism [5], precisely because rigorous demonstration of synchrony is difficult, especially in the absence of experimental data. In 1968, Buck & Buck [6] provided photometric evidence to demonstrate the occurrence of synchrony in *Pteroptyx malaccae* congregations in Thailand. In 1983, Cicero [7] published a thorough account of lek behaviour in the Arizona firefly *Photinus knulli*, following extensive observations made by eye. These observations did include the occurrence of synchrony. Beginning in the early 1990s, Eastern US synchronizing counterparts *Photinus carolinus* and *Photuris frontalis* have received ample attention from scientists [8], bringing videographic evidence to characterize the occurrence [9,10], mechanisms [11,12], possible function [13,14] and modalities of synchronous patterns [15]. In parallel, this renewed interest in firefly synchronization motivated numerous developments of mathematical and computational models [16–18].

In prior experimental studies, owing to the very high number of fireflies flashing in unison, the occurrence of synchrony was simply accepted from observations and raw data, without demanding further statistical analysis. The high signal-to-noise ratio was sufficient proof. Since then, recurrent speculation about possible synchrony in other species illustrates the need for a general methodology, adapted in particular to low-density populations.

This paper proposes to address the following question: How does one characterize synchronization behaviour among several dispersed fireflies? The situation at hand is different from other typical situations where synchrony is involved. In mathematical models and numerical simulations, where each agent’s internal phase *θ*_*k*_(*t*) is known at all times, it is possible to calculate a phase-coherence order parameter, $R(t)=|{\left\langle e^{i\theta_{k}}\right\rangle }_{k}|$, whose value between 0 and 1 quantifies the degree of synchrony within the system [19]. In situations where only firing is detectable, but each agent is continuously tractable, such as systems of neurons, it is possible to calculate cross-correlations between firing times of different constituents [20]. For fireflies, however, their internal phase is unknown, and in their natural habitat individual fireflies cannot be tracked for more than the duration of a flash train, after which they vanish into obscurity. We propose here two approaches to demonstrate synchrony from high-resolution video recordings: (i) from the time series of the number of flashes in a camera’s field of view; and (ii) from the spatio-temporal correlations between flash occurrences, after three-dimensional reconstruction of the swarm.

## 2. Methods

**Figure 1.**
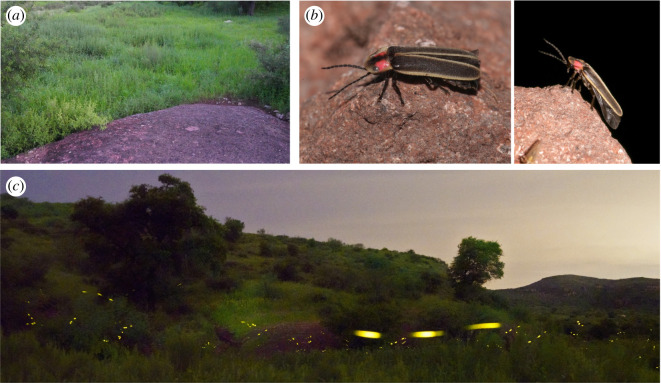
(*a*) Field of view from one of the two recording cameras, near the river bed of the Peña Blanca Canyon. (*b*) *Photinus knulli* male firefly. (*c*) Long exposure (15 s) photograph of a collective display of *P. knulli* near the recording site. Several flash triplets are apparent.

The general experimental area was the same as described in detail in [7], namely the Peña Blanca Canyon (above lake) within Coronado National Forest (Pajarito Mountains, Santa Cruz County, AZ, USA). It comprised an intermittent river bed with a gravel road and nearby campground. The area where video recordings occurred was situated on the side of the canyon opposite from the road and covered with dense vegetation (figure 1*a*).

Field observations occurred every night between 4 August and 13 August 2021. The first flashes could generally be seen between 19.50 and 20.00 local time (Mountain Standard Time, MST). Sunset occurred around 19.15 MST. Temperatures were in the 20–25°C range. Temperature and humidity variations were consistent and moderate because of clear or overcast evenings but within parameters characteristic of monsoon season in the Arizona Sonoran Desert Sky Island habitat of the Pajarito Mountain range.

Owing to initial adjustments and varying environmental conditions (rain, transient flooding), three full datasets of stereoscopic recordings were eventually obtained (7, 9, 10 August, about 150 min each). Recordings were obtained as described previously [15] from two Sony *α*7R4 cameras set at 60 frames per second (fps) and mounted with a wide-angle lens. Hence, the time resolution of our experiments was 17 ms. Video processing and data analysis were performed with Matlab. Flash centroids were extracted by intensity thresholding after background subtraction. For stereoscopic recordings, camera pairs were calibrated using about 10 pairs of pictures of a chessboard (25 cm side length) and Matlab’s stereoCalibration toolbox. Flash three-dimensional positions were subsequently obtained by triangulation. Spatial resolution is typically in the 1 mm–10 cm range for flashes occurring within 30 m from the cameras [12].

In order to confirm species identification, a few specimens were collected every night with an insect net and carefully inspected, then released. Some were photographed as well.

## 3. Results

### 3.1. Preliminary observations

#### 3.1.1. Species identification

Collected male individuals (figure 1*b*), either on the ground or in flight, were all consistent with previous descriptions of *P. knulli* morphology [7]. From collected specimens and general visual observations, it appeared that only one species was displaying (flashing) over the course of our field experiments. Of note, however, glowing larvae, identified based on direct rearing as possibly *Pleotomus nigripennis* LeConte, were also observed in the leaf litter and on tree trunks.

#### 3.1.2. Individual flashing

By eye and in close proximity, *P. knulli* flying males appear to emit flash triplets spanning a period of about 1s (figure 1*c*). This was confirmed by video recordings (see electronic supplementary material, figure S1). Occasionally, flash phrases of two, four or five flashes can also be observed, but are much less common. Flash triplets from a single individual are typically separated by at least 3–5s. Males were dispersed and solitary in their emergence.

### 3.2. Time series reveal spikes of correlated activity

**Figure 2.**
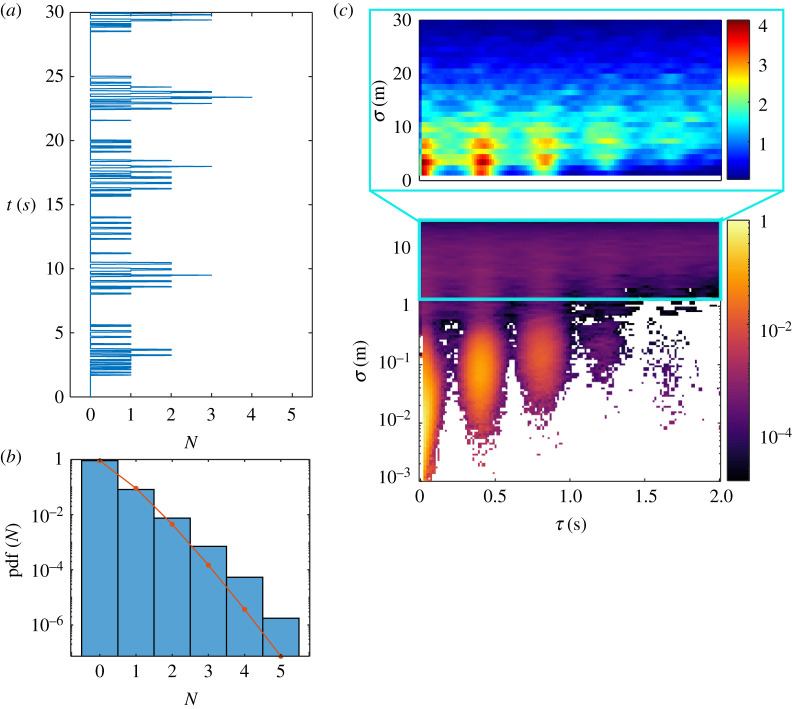
(*a*) Sample time series of the number *N* of flashes per frame. For visibility, only a short 30 s interval is shown. Many concurrent flashes happen during repeated bursts of activity. (*b*) Experimental probability distribution (pdf) of *N* from over 2 h of data (10 August). Red line is the result for a Poisson distribution with the same average *λ* as pdf (*N*). (*c*) Spatio-temporal correlations: distribution of separation *σ* and time delay *τ* between flash occurrences (150 min of data from 10 August). The bottom plot shows the full range of spatial separations, dominated by self-correlations at short range (*σ* < 1 m). The top plot shows the distribution only for *σ* > 1 m, emphasizing extrinsic correlations among distinct fireflies. The colour schemes indicate the relative frequency of different (*τ*, *σ*) domains.

To analyse patterns of collective flashing in *P. knulli*, we first calculated the number *N* of flashes detected in each movie frame (figure 2*a*). Since firefly activity was relatively low, most frames contained no flash, some captured one single flash and a few captured between two and five flashes, under the given experimental conditions. (Owing to the cameras’ limited field of view and light sensitivity, and visual obstruction from the bottom vegetation, recorded flashes account for only a fraction of the swarm’s total.) While synchrony implies that several flashes occur at the same time, the reverse proposition is not necessarily true: concurrent flashes could happen by accident, even with no underlying interactions between fireflies. Intuitively, however, if the proportion of concurrent flashes is large, it tends to indicate intrinsic correlations. To quantitatively evaluate this proposition, we must compare the observed distribution of flashes *P*(*N*) against the null hypothesis that flashes happen independently at random. Independent events happening within a given time frame at a constant rate *λ* are described by the Poisson distribution: Poiss(*N* = *k*) = *λ*^*k*^ e^−*λ*^/*k*!. In our situation, because the swarm’s flashing rate was fairly constant around a mean value of *λ* = 0.099 flashes/frame (see electronic supplementary material, figure S4), we can compare the experimental distribution *P*(*N*) with the Poisson distribution with the same average, as shown in figure 2*b*. Evidently, the proportion of large values of *N* is vastly superior (about one order of magnitude) to what would be predicted from the Poisson distribution, hinting that concurrent flashes are too frequent to occur only by chance. To quantify this discrepancy, we employed goodness-of-fit tests for the Poisson distribution. Several such tests have been developed [21]. For their simplicity of implementation and interpretation, we used two test statistics, *Z* and *T*, based respectively on the first and second, and third and fourth, moments of the empirical distributions [21,22]. As a brief reminder, the Poisson distribution has mean equal to variance and squared-skewness equal to excess kurtosis. Significant deviations from these equalities indicate non-Poissonity. Owing to appropriate normalizations, both *Z* and *T* are asymptotically distributed according to a normal distribution of mean 0 and variance 1 (see electronic supplementary material, figure S2). For the empirical distribution of *N* in figure 2*b*, we find *Z* = 55 and *T* = −28, which significantly deviate from the range of values expected if the underlying distribution were Poisson (corresponding *p*-values are *p*_*Z*_ ∼ 10^−67^ and *p*_*T*_ ∼ 10^−18^; see electronic supplementary material, §4). Therefore, the null hypothesis is rejected by the test statistics, at a very high significance level. Collective flashes are not independent, and *P. knulli*’s collective display can be considered synchronous with extremely high probability, at least episodically. Similar conclusions can be drawn from the time series from the two other datasets (see electronic supplementary material, figure S4).

The validity of the Poisson distribution for the null hypothesis here should be nuanced. Indeed, flashes typically span not just one frame, but up to six, and many are repeated three times within a short period. Therefore, the distribution of *N* contains internal correlations, even if flashers were fully independent. However, we show in the electronic supplementary material that persistent, repeated flashing from independent agents does not significantly alter the resulting distribution of test statistics from one-flash-per-frame distributions, hence the comparison with the Poisson distribution still holds.

### 3.3. Spatio-temporal distributions reveal interactions

Synchronous behaviour was also made apparent from the distribution of spatio-temporal correlations between flash occurrences. For two three-dimensionally reconstructed flashes of coordinates (*x*_*i*_, *y*_*i*_, *z*_*i*_, *t*_*i*_) and (*x*_*j*_, *y*_*j*_, *z*_*j*_, *t*_*j*_), we can calculate the corresponding separation *σ*_*ij*_ = |(*x*_*j*_, *y*_*j*_, *z*_*j*_) − (*x*_*i*_, *y*_*i*_, *z*_*i*_)| and time delay *τ*_*ij*_ = |*t*_*j*_ − *t*_*i*_|. Pairs of relative coordinates (*τ*, *σ*) are independent from a particular frame of reference, hence all data can be collapsed onto the same plot. They inform about how flashes self-organize over time- and translation-invariant patterns. Peaks in their distribution indicate recurrent relationships between localization and timing, which reveals underlying correlations and propagation of information, as previously studied in other species [12].

The two-dimensional distribution of (*τ*, *σ*) for *P. knulli* is presented in figure 2*c*. Several features are significant. For *σ* < 1 m, the correlations are narrowly distributed around three peaks at *τ* ≃ 0, 0.45, 0.9 s. These correspond to the flash triplets emitted by individual fireflies, which remain localized as they travelled typically no more than 0.5–1 m over 1 s. Owing to the swarm’s low density, flashes from different fireflies happening within 1m from each other were very rare. Next, secondary peaks are visible for *σ* > 1 m. These cannot originate from the same firefly, since it would not travel fast enough. They therefore represent extrinsic correlations. The fact that these peaks are narrow and distributed at the same specific times as intrinsic correlations is further proof of synchrony. Importantly, synchronizing respondents were primarily situated in the 1–10 m range, as correlations vanish at larger distances. This is in line with previous findings that synchronizing information propagates only at short range [15].

## 4. Discussion and conclusion

Remarkably, the collective flashing pattern of *P. knulli* appears to mirror that of another species of the same genus, *P. carolinus*. Both species flash synchronously during bursts of activity lasting a few seconds and repeated periodically, as evidenced by the frequency spectra of their respective time series (see electronic supplementary material, figure S5). Collective bursts are typically repeated every 12–14 s for *P. carolinus*, but only every 5 s for *P. knulli*. This difference seems most likely attributable to the individual flash phrase, consisting of three flashes for *P. knulli*, but six to eight for *P. carolinus*. Spatio-temporal correlations between flash occurrences further emphasize this distinction, but also show a similar spatial range of interaction (see electronic supplementary material, figure S5). These qualitative similarities but quantitative differences offer an interesting opportunity to test mathematical models of intermittent synchrony.

Overall, our video recordings largely confirm the observations made in [7], and provide data and statistical analysis to demonstrate the synchronous behaviour of *P. knulli*. While our analysis confirms the occurrence of synchrony in this third North American species, the absence of enough firefly density prohibited the observations of more complex behaviours previously reported in [7]. It follows from a notable drop in firefly activity in the Peña Blanca Canyon over the past three decades. This population decline is concerning [23]. While fireflies exist across the American West, populations tend to be much more sparse and localized than in the East. These swarms are rare and fragile; *P. knulli* is now considered as Vulnerable by the International Union for Conservation of Nature [24]. Over the past few decades, increased habitat degradation (e.g. increased ranching and illegal all-terrain vehicle recreation) and changing weather patterns may be putting populations and even entire species at risk. While 2021 was an excellent monsoon season in southern Arizona, prior years had been excessively dry. Western populations have also been traditionally less studied and less monitored than eastern ones; over 50% of species are considered data deficient. Continued monitoring and research efforts are important to further understand population dynamics. In the meantime, it seems crucial to encourage local initiatives to further protect firefly habitats.

## Data Availability

Datasets of three-dimensional reconstructed flash occurrences for 7, 9, 10 August 2021 are made available as part of the electronic supplementary material [25].
